# Burst predicting neurons survive an *in vitro* glutamate injury model of cerebral ischemia

**DOI:** 10.1038/srep17718

**Published:** 2015-12-09

**Authors:** Eric S. Kuebler, Joseph S. Tauskela, Amy Aylsworth, Xigeng Zhao, Jean-Philippe Thivierge

**Affiliations:** 1 Center for Neural Dynamics and School of Psychology, University of Ottawa, Ottawa, Ontario, Canada; 2 Department of Translational Bioscience, Human Health Therapeutics, National Research Council of Canada, Ottawa, Ontario, Canada

## Abstract

Neuronal activity *in vitro* exhibits network bursts characterized by brief periods of increased spike rates. Recent work shows that a subpopulation of neurons reliably predicts the occurrence of network bursts. Here, we examined the role of burst predictors in cultures undergoing an *in vitro* model of cerebral ischemia. Dissociated primary cortical neurons were plated on multielectrode arrays and spontaneous activity was recorded at 17 days *in vitro* (DIV). This activity was characterized by neuronal avalanches where burst statistics followed a power law. We identified burst predictors as channels that consistently fired immediately prior to network bursts. The timing of these predictors relative to bursts followed a skewed distribution that differed sharply from a null model based on branching ratio. A portion of cultures were subjected to an excitotoxic insult (DIV 18). Propidium iodine and fluorescence imaging confirmed cell death in these cultures. While the insult did not alter the distribution of avalanches, it resulted in alterations in overall spike rates. Burst predictors, however, maintained baseline levels of activity. The resilience of burst predictors following excitotoxic insult suggests a key role of these units in maintaining network activity following injury, with implications for the selective effects of ischemia in the brain.

Network bursts are a common feature of several *in vitro* preparations of the central nervous system. They are characterized by short transients of rapid spiking activity flanked by silent periods. Cultures of cortical neurons begin to emit network bursts around day *in vitro* (DIV) 6 and gradually develop a rich repertoire of activity during the course of maturation^1^. Experimental work has associated network bursts with the propagation of neuronal information^2^,^3^ and the formation of central pattern generators^4^. The statistics of spontaneous network bursts follow a power law distribution, with scaling exponents that are suggestive of a critical state that promotes information processing^5^ (with some controversy surrounding the issue^6^).

Experimental work has characterized subsets of neurons that reliably predict the occurrence of network bursts^7^,^8^. Both experimental and theoretical work suggests that these burst-predicting cells have stable long-term spiking dynamics that can be modified by electrical stimulation^9^,^10^. Cells that predict bursts of activity may therefore be central to network function.

Because burst-predicting cells play a causal role in the initiation of network activity^11^, their survival following a neural injury may be crucial to the maintenance of network bursts. In hippocampus, burst predicting cells have been identified as GABAergic interneurons with long-range (or short-range, but dense) axonal arborisation^11^. These cells are more resistant than principal (pyramidal) cells to an ischemic insult^12^, suggesting that burst predictors may indeed be less susceptible to neurotoxic insult. This link, however, has to our knowledge never been established directly.

Here, we examined how *in vitro* network activity is altered by an excitotoxic glutamate insult that captures key features of oxygen-glucose deprivation during ischemia^13^. Glutamate is a neurotransmitter found in great abundance in the central nervous system and has been associated with long-term potentiation by binding with and activating both NMDA and AMPA receptors on post-synaptic sites^14^. In this state, neurons can suffer from an over-abundance of glutamate in the synaptic cleft, and be constantly excited, in turn succumbing to excitotoxicity.

We cultured dissociated cortical cells on multielectrode arrays (MEAs, Fig. 1a) and extracted multiunit activity (MUA) from each electrode (Fig. 1b), which reflects the combined spiking activity of neurons surrounding the electrode^13^,^15^,^16^,^17^. We examined baseline neuronal activity at DIV 17 to identify channels that predict network bursts (Fig. 1c). To assess the viability of burst predicting cells, a portion of the cultures were subjected to a glutamate insult, where on DIV 18 we added ouabain-TBOA to the extracellular fluid. While the ischemic insult caused marked cell death, both neuronal avalanches and burst predictor firing rates were largely maintained. These findings have implications for the functional recovery of neuronal networks following cerebral ischemia.

## Results

### Impact of Glutamate Injury on Neuronal Cultures

We first evaluated the percentage of death caused by 10 min (‘low’ kill) and 20 min (‘high’ kill) exposures to ouabain-TBOA insults applied at 18 DIV, as determined by the ratio between the number of dead (Fig. 2a, bottom row, PI-positive neurons measured 1-day post-insult at 19 DIV) and live neurons (Fig. 2a, top row, phase-bright neurons measured immediately prior to the insult at 18 DIV). Representative same-field phase contrast and PI fluorescence images were acquired before and after 20 min ouabain/TBOA (Fig. 2a). Control cultures (i.e., without insult) displayed low basal neurotoxicity, indicating high quality neuronal cultures. By comparison, the 10 and 20 min ouabain/TBOA insults yielded successive increases in neurotoxicity (Fig. 2b).

### Impact of Injury on Neuronal Activity

#### Avalanches

Network bursts *in vitro* are often characterized by avalanches in which smaller (or shorter) events occurred more often than larger (or longer) events^18^,^19^. We measured the propagation of activity in both baseline and post-insult cultures by examining three distributions: (1) the duration of avalanches; (2) the total number of cells activated at least once during avalanches; and, (3) the number of MUA events generated during avalanches. Our results show that baseline distributions of all three measures were approximately linear in log-log coordinates, suggesting a power law distribution (Fig. 3). Consistent with previous work, we estimated the slope of power laws using a maximum likelihood method^17^,^18^. The estimated slope 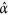 for a bounded discrete power law follows


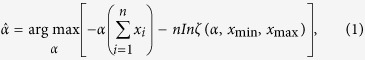


where *x*_*i*_ is the *i*th element from the vector of all data *X* = [*x*_*1*_, *x*_*2*_, …, *x*_*n*_] and *n* is the size of the data vector. Techniques for estimating lower and upper bounds from raw data are covered elsewhere^20^; here, we set *x*_*min*_ and *x*_*max*_ to the minimum and maximum values observed in the data. The *n*th element of *X* corresponds to the duration of time (measured in ms), number of units active during the *n*th avalanche recorded, and the number of MUA events (Fig. 3, left, middle, and right panels, respectively). The Hurwitz zeta function, 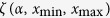 is given by


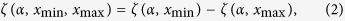


where


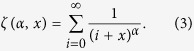


Consistent with previous work^17^,^18^, the estimated slope 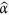 of all power law distributions was near 1.5 (albeit slightly above, see Fig. 3, coloured insets). Although the insult yielded marked cell death (Fig. 2), the propagation of activity was largely maintained, with a slight drop in power law exponents for low kill as well as high kill MEAs (Fig. 3). The presence of avalanches in the activity of post-insult cultures implies that spontaneous neuronal dynamics were largely intact despite high levels of neuronal death.

We compared these results with those obtained from a branching model^19^ that produces avalanches of network bursts. First, we computed the critical branching parameter for all baseline recordings. This branching parameter (*σ*) quantifies the ratio of downstream electrodes that become active as a function of upstream activation. The branching parameter is computed by


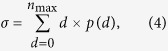


where *p(d)* is the probability of observing *d* descendants, and *n*_*max*_ is the maximal number of active electrodes. Descendants *d* are computed by


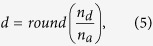


where *n*_*a*_ is the number of ancestors observed in the first time bin, *n*_*d*_ is the number of active electrodes in the second time bin, and *round* is the rounding operator to the nearest integer^15^,^19^. The probability of observing *d* descendants was computed by


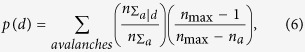


where 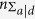 is the total number of ancestors when *n*_*d*_ descendants were observed, 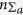 is the total number of ancestors observed, and


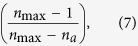


is a factor that provides a correction for the number of electrodes available in the next time bin because of refractoriness^19^. Mean propagation was *σ* = 0.97 (s.d., 0.04). This means that on average, every event was followed by ~1 event, as consistent with a 3/2 slope of avalanches^15^.

Next, we paired the result of the above critical branching analysis with a propagation model. We designed a randomly connected network of *N* = 100 binary (on or off) units that made *C* = *N* synapses (all-to-all connectivity). All synapses had a probability *p*_*i*_ of propagation that was randomly chosen, but constrained so that the sum of all presynaptic connections was equal to


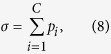


where 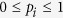 and 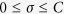. With *σ* = 1, the model generated irregular activity with transients of network synchronization similar to experimental data (Fig. 4b, black). The best-fitting slope for avalanche activity (number of spikes) was α = 1.5. Increasing the branching parameter (*σ* = 1.2) resulted in larger network bursts (Fig. 4b, red). As a result, the best-fitting slope was lower than 1.5 (α = 1.3). Conversely, decreasing the branching parameter (*σ* = 0.8) led to smaller network bursts (Fig. 4b, green) whose distribution was fitted with a steeper slope (α = 1.7).

### Impact of Injury on Burst Predictors

#### Burst predictors

**Figure 1:**
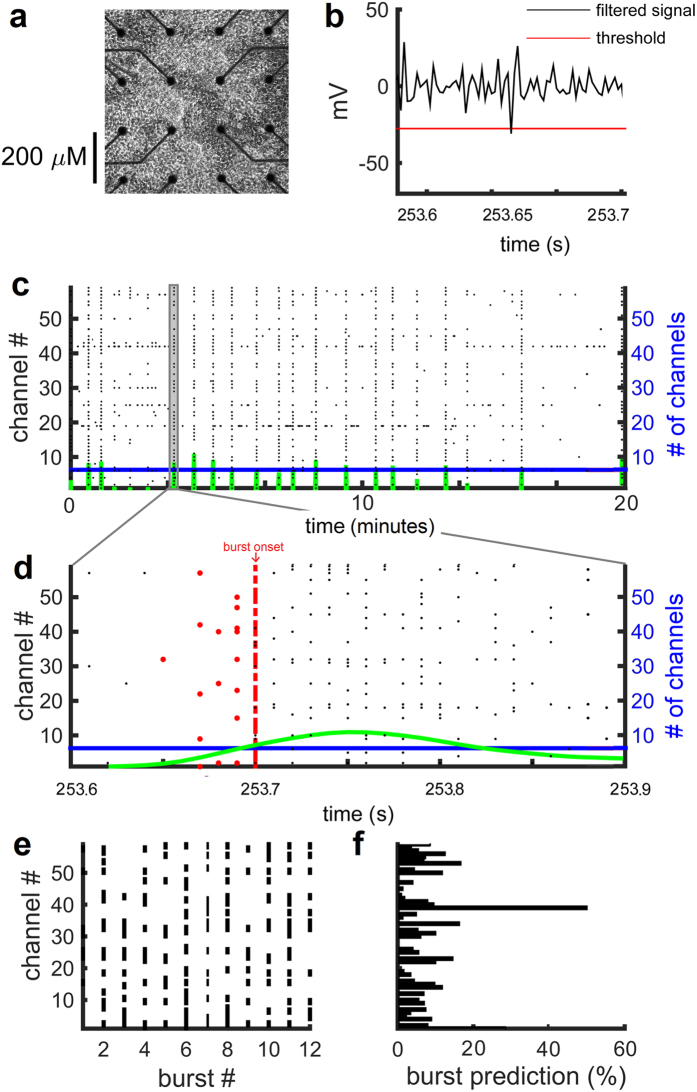
*In vitro* network bursts on multielectrode arrays. (**a**) A subset of electrodes on a multielectrode array (MEA) imaged under phase contrast conditions to allow the discernment of neuronal somas. The 4 × 4 grid shown here was located in the center of the cell culture. (**b)** Typical recording of multi-unit activity (MUA) surrounding an electrode (high-pass filtered at 200 Hz). The red trace displays the MUA detection threshold for this channel. (**c**) Raster plot showing MUA events recorded at each channel for the entire 20 min recording. Grey shaded area is zoomed in below. (**d**) Same as (**c**) but zoomed in to show a single network burst (~300 ms). Red circles indicate electrodes identified as burst predictors (i.e., electrodes that spiked <50 ms before a network burst). Green, average population activity. Blue, threshold for the detection of network bursts (see Methods). (**e)** Raster plot showing electrodes that recorded at least one MUA event before a burst in (**c)**. (**f**) Burst prediction scores for the data in (**e**).

To further characterize the impact of ischemic injury on spontaneous neuronal activity, we identified electrodes that reliably predicted network bursts (i.e., Fig. 1d). We computed a burst prediction (BP) score for each channel at baseline (DIV 17) that measures the propensity of a given channel to be active immediately prior to a network burst (within a pre-burst window of 50 ms), normalized by the mean firing rate (see Methods). The distribution of BP scores was positively skewed, with a heavy tail towards higher values, suggesting that a small subset of electrodes were responsible for a large proportion of BP scores (Fig. 4a).

By comparison, BP scores obtained with the branching model were low and followed a narrow distribution (Fig. 4c). This is explained by the random propagation of activity in this model, and therefore serves as a null model against which experimental scores may be compared. The marked difference in BP scores between the model and experiments suggests that strong burst predictors do not emerge naturally from a process of random propagation.

Are the BP scores obtained from cortical recordings related to firing rates? Plotting BP scores against firing rates reveals that the highest burst predictors (i.e., values greater than 20) had low firing rates (<0.5 Hz). Conversely, lower burst predictors (values below 20) had a broad range of firing rates, including both higher (>0.5 Hz) and lower (<0.5 Hz) values. Overall, firing rates were not strongly correlated with BP scores (Pearson correlation, *r*^2^(413) = −0.0004, *p* > 0.372).

#### Alterations in the Distribution of BP Scores

Was the distribution of BP altered following ouabain-TBOA treatment? To answer that question, we compared burst prediction scores before and after treatment in low and high kill conditions. The overall distribution of scores remained similar at DIV 17 (pre-treatment) vs. 21 (post-treatment) for low kill (Wilcoxon rank test, *z* = 1.62, *p* > 0.106) as well as high kill conditions (*z* = 1.14, *p* > 0.256). Therefore, overall distributions of burst prediction scores were not significantly altered by ouabain-TBOA treatment.

In addition, we investigated whether BP scores diminished in accordance with the ratio of cell death. We examined this question by calculating the correlation between two values, namely (*i*) the percentage of electrodes that no longer emitted spikes following treatment; and (*ii*) the mean burst prediction score across all electrodes. This correlation was not significant (*r*(21) = 0.35, *p* > 0.124; low-kill and high-kill conditions were pooled), indicating that mean burst prediction scores did not relate straightforwardly to the percentage of inactive electrodes following ouabain-TBOA treatment.

#### Alterations in Firing Rate

To determine whether individual burst predictors were susceptible to insult, we examined the difference in firing rate (*Δrate*) between baseline and post-insult recordings across single channels (Fig. 5a). For each channel of a given MEA, we subtracted the mean firing rate at baseline (DIV 17) from the mean firing rate at DIV 21. Values of *Δrate* that fall below zero indicate a post-insult decrease in firing rate. Across each condition, strong burst predictors (BP scores greater than 20) maintained firing rates that were comparable to baseline. By comparison, weak burst predictors (BP scores lower than 20) in the control and low kill conditions showed mainly positive (and some slight negative) changes in firing rate. In the high kill condition, on the other hand, weak burst predictors showed mainly negative changes in rate. Baseline firing rates were not strongly related to *Δrate* values (*r*^2^(413) = 0.02, *p* > 0.002). In sum, a small subset of strong burst predictors displayed firing rates that were highly resistant to changes following the excitotoxic injury.

Was the identity of strong burst predicting cells the same before and after treatment? We addressed this question by grouping electrodes in the low kill condition according to their pre-treatment (DIV 17) burst predicton scores. Electrodes with the top five scores (“high BP” group) retained significantly higher scores after treatment than all other electrodes (“low BP” group) (paired t-test, *t*(20) = −2.36, *p* < 0.028) (Fig. 5b). Therefore, electrodes with high burst prediction scores before treatment had a strong tendency to maintain (or increase) high scores following treatment.

The stability of burst prediction scores over several recordings was further demonstrated using a principal components analysis of these scores across five days *in vitro* (DIV 15, 16, 17, 21 and 22). The largest principal component (accounting for 55% of the variance) was strongly related to mean burst prediction scores (Fig. 5d), showing a consistency of mean scores across recordings.

The stability of strong burst predicting cells does not, however, preclude the possibility that cells identified as low predictors before treatment emerge as strong predictors following treatment. To investigate this possibility, we identified electrodes with a score of zero during pre-treatment recordings (DIV 17) in the low kill condition. For a portion of these electrodes, post-treatment (DIV 21) scores were greater than zero, indicating the emergence of new burst predictors following treatment (Fig. 5c). These results suggest a reorganization of network activity following ouabain-TBOA insult.

Overall, results show that the activity of strong burst predicting cells remained stable despite some degree of network reorganization following neuronal insult. Next, we discuss the implications of these results for our understanding of the links between network activity and ischemic injury.

## Discussion

The aim of the current study was to examine whether cells that predicted the occurrence of network bursts were more (or less) susceptible than others to an *in vitro* model of ischemia. We computed BP scores that reflected the ability of individual channels to predict upcoming network bursts. Overall, these scores showed a highly skewed distribution that could not be reproduced by a branching model with random activation sites. Further, BP scores could not be accounted for strictly on the basis of firing rates, changes in propagation delays, or spatial clustering on the arrays (see Supplementary Material). Following an excitotoxic insult, we found that channels with high BP scores (i.e., strong burst predictors) maintained firing rates that were comparable to baseline, while weak burst predictors showed marked alterations (either an decrease or increase) in activity. Together, results highlight the resilience of strong burst predictors to an excitotoxic insult, and open the possibility that these units contribute to the maintenance of network activity following ischemia.

The idea that neuronal injury may target different aspects of brain networks while leaving other aspects intact has been examined across a broad range of studies. In adult mice, for instance, the rabies virus may be selectively taken up by sensory neurons and then distributed to the dorsal root ganglia^21^. In humans, different subgroups of neurons have a distinct immune signature that contributes to susceptibility to infection^22^. This differential susceptibility may have consequences (albeit largely unknown) on information processing in local networks^23^.

It remains unclear what might explain the resilience of strong burst predictors to an ischemic insult. In hippocampus, strong burst predictors have been identified as GABAergic interneurons with axonal arborisations that are either long-range or short and dense^11^. In several brain regions, GABAergic interneurons are more resistant to cell death than principal neurons^12^. Functional studies, however, show that ischemia impacts the activation of GABAergic neurons through a rise in CI^−^ that reduces the CI^−^ influx of these cells^12^. Further work is required to resolve this question and examine the role of GABA transmission in strong burst predictors both *in vivo* and *in vitro* as well as their link to ischemia.

One question we did not examine here is the possibility of a delayed effect of ischemia on strong burst predictors. Because our recordings were limited to three days post-insult, it is possible that we did not detect effects of ischemia that occur over a longer time period. Future work that extends our results by recording activity several days *in vitro* following excitotoxic insult would allow us to clarify whether network activity (and particularly burst predictors) remains stable across a longer time period. Finally, it is unclear from our results whether burst predictors merely anticipate network bursts or whether they drive their occurrence in a causal fashion^11^. Addressing this question is technically challenging, as it requires the identification of strong burst predictors during ongoing recordings. This would constitute an important step in understanding the relationship between burst predictors, network activity, and ischemic injury.

## Methods

### Cultured Neurons on Multi Electrode Arrays

All experiments were approved by the Human Health Therapeutics Animal Care Committee at the National Research Council Canada and carried out in accordance with approved guidelines. Culturing and plating of primary cortical/hippocampal neurons was performed as previously described^16^. Briefly, time-pregnant embryonic day 18 Sprague-Dawley rats (Charles River, St. Constant, Quebec, Canada) were anesthetized with halothane and culled by cervical dislocation. Following dissection of the cortical and hippocampal regions of the fetal brains, cells were centrifuged at 1,000 *g* for 3 min at 4 °C and were dispersed by gentle trituration. Cells were plated on MEAs at a high density of 1.8 × 10^6^ cells/ml of medium (consisting of EMEM (Wisent) supplemented to 25 mM glucose), 10% fetal bovine serum (PAA), 10% horse serum (Sigma-Aldrich, St Louis, U.S.A.), and pen/strep (Gibco 1X). The entire surface of each MEA dish (Multi-Channel Systems, Reutlingen, Germany) was pre-treated with a high molecular weight poly-L-lysine (0.025 mg/mL diluted in 1×PBS; Sigma-Aldrich) and laminin (0.02 mg/ml; Gibco) to promote cell adhesion and minimize cell migration. To better approximate neurotoxic insult conditions previously employed in our laboratory, cell suspensions were plated at 1 ml/MEA so that the entire MEA surface (and not just the inner electrode region) was coated with cells^13^,^16^. Cultures were maintained in a humidified incubator at 37 °C with a 5% carbon dioxide / 95% air atmosphere. Osmolality was strictly controlled by daily addition of distilled water and by covering MEAs with a transparent, gas-permeable polydimethylsiloxane lid to prevent evaporation^24^. To minimize glial cell proliferation, a mitotic inhibitor (15 μg/ml of 5-fluoro-2′-deoxyuridine and 35 μg/ml of uridine) was added at 4 *days in vitro* (DIV). In addition, MEAs were gently rinsed and media was filtered starting at 7 DIV and repeated every 4 days. A 50% media (containing 10% horse serum but not fetal calf serum) change was performed at 7 DIV, with 33% media changed every 3 DIV thereafter.

### Recordings of Spontaneous Neuronal Activity

Immediately prior to a recording session, an MEA was removed from the maintenance incubator, capped with a sterile vented lid and placed in the acquisition platform housed within a 37 °C incubator. A 20 min equilibration period allowed for minimization of movement-stimulated activity. Spontaneous activity was recorded in 20 min sessions at 17–18 and 21–22 DIV using MC Rack software (Multi-Channel Systems) employing the following settings: unit-less amplifier gain (1100.0), input voltage range (+/− 2048 mV) and acquisition rate (5 kHz).

Data from the multi-unit recordings were analyzed offline using custom software written in MATLAB (Mathworks Inc., Natick, Massachusetts, USA). We preprocessed the raw voltage recordings as in previous work^15^,^16^. First, each channel’s activity was down-sampled from 5 kHz (acquisition rate) to 1 kHz. Raw voltages were then stored in a matrix **X** of size *N* by *T*, where *N* = 59 is the number of electrodes analyzed and *T* = 1,114,000 ms is the number of milliseconds in a single recording session (20 minutes). We applied a 2^nd^ order high-pass Butterworth filter with a cut-off frequency of 200 Hz to retain high frequency deflections (Fig. 1b, black trace). Consistent with related work^25^,^26^, multi-unit activity (MUA) was detected by setting a threshold 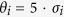 (Fig. 1b, red line) for a given channel *i*, where


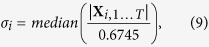


where the matrix **X** contains bandpass filtered signals, each row is a channel indexed by *i*, and each column is a time-step (measured in ms). For each millisecond time-step, activation was detected when the *i*th channel’s voltage was lower than or equal to threshold 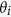. This process was repeated for all electrodes generating a matrix **Y** of the same size as **X**, with ‘1’s and ‘0’s denoting activation or silence, respectively (Fig. 1c). Channels with fewer than two MUA events or firing rates greater than five standard deviations away from the mean were removed.

### Burst Predictors

We sought to characterize the ability of different units to predict the upcoming occurrence of network bursts (Fig. 1d). We first downsampled the recorded data from 1 kHz recordings by a factor of 10. This operation resulted in a matrix **Z** of size *N* by *T*/*B*, where *B* = 10 ms, with 1’s and ‘0’s denoting the presence or absence of at least one MUA event, respectively, in a given bin of 10 ms. To detect network bursts, we summed network firing activity across channels, and employed a 100-fold bootstrapping method where the original data was compared to randomly permuted data. For each random matrix, the firing rate of each channel was maintained; the firing times, however, were randomly permuted. This random matrix was summed across channels, producing a vector that was compared to the experimental data. Times when the experimental data exceeded 95% of the random vectors were marked as bursts (Fig. 1c-d, blue line marks the 95% threshold). Cultures with less than 5 bursts for an entire baseline recording were removed from further analysis (12 out of 42 total cultures were removed). Finally, we computed a burst prediction (BP) score for each channel (Fig. 4f). This score is computed by counting the number of network bursts preceded by at least one MUA event by at most 50 ms (Fig. 1d, red dots), and dividing by the total number of network bursts. The resulting score was divided by the mean rate of each channel to control for electrodes with high firing rates that were not specific to network bursts.

### Neurotoxic Insult

Following baseline recording sessions (17 DIV), cultures at 18 DIV were exposed to a combination of a Na^+^/K^+^ ATPase inhibitor, 5 μM ouabain (Sigma-Aldrich, St. Louis, Missouri, USA), with a glutamate uptake inhibitor, 40 μM DL-threo-β-benzyloxyaspartic acid (TBOA; Tocris Bioscience, U.S.A.), for 15 min (‘low’ kill) or 20 min (‘high’ kill) or to the DMSO vehicle for 20 min (control). A combined concentration of ouabain and TBOA was added via pipette directly to the extracellular fluid of cultured cells in the insult condition. Ouabain inhibits the sodium-potassium pump (or Na^+^/K^+^ -ATPase), and reduces the amount of both sodium pumped out and potassium pumped into the cells^27^. TBOA is a glutamate transport inhibitor that prevents cellular uptake of glutamate thereby increasing the extracellular concentration of neurotransmitters^28^. We have previously shown that this insult selectively kills neurons by an excitotoxic mechanism, specifically the build-up of extracellular glutamate causing neurotoxic activation of postsynaptic NMDA receptors^29^. The ouabain-TBOA model of stroke causes overexcitation throughout the neuronal network to the point of toxicity, and in some cases cell death.

### Assessment of Neuronal Injury

**Figure 2:**
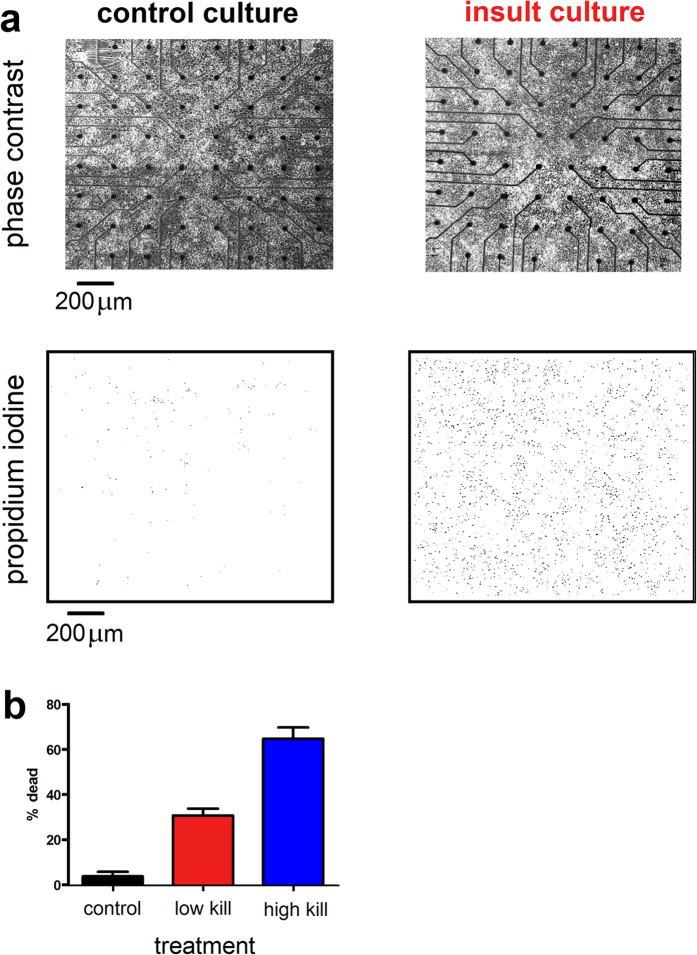
Excitotoxic insult causes widespread neuronal death. (**a**) Two representative phase contrasts (top row) as well as propidium iodide (PI) and fluorescent images (bottom row). PI images have inversed colour tones. (**b**) Percentage of dead neurons for each treatment condition (see Assessment of Neuronal Injury section). Error bars are standard error of the mean.

**Figure 3:**
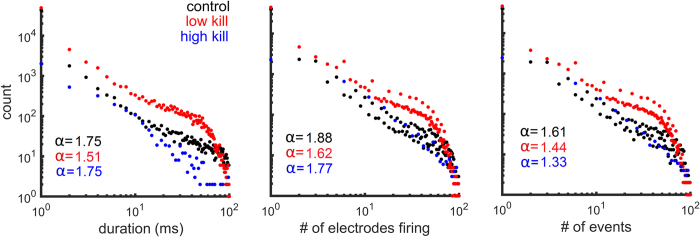
Impact of insult on neuronal avalanches. *Left panel*: histogram in log-log coordinates of the duration of avalanches. *Middle panel*: number of electrodes active during an avalanche. *Right panel*: number of MUA events. Maximum likelihood estimates of power law slopes (α) for each distribution are shown on each plot. Control data correspond to DIV 17 and both low kill and high kill data correspond to DIV 21.

**Figure 4:**
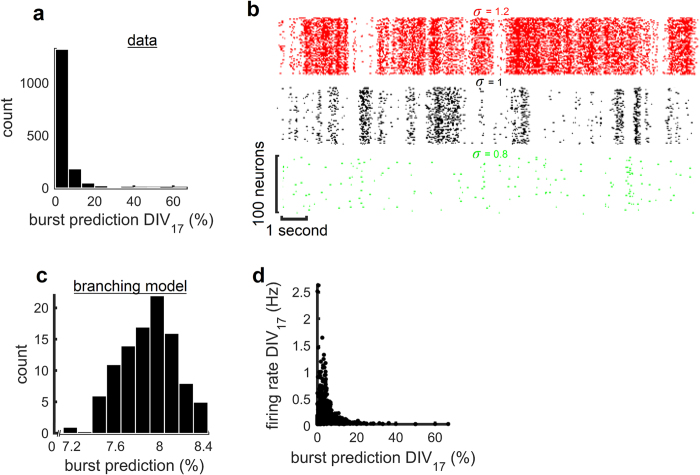
Burst predictors and firing activity. (**a)** Histogram of burst predictor scores for all cultures at baseline (DIV 17). (**b**) Spike raster depicting network activity generated by the branching model (*N* = 100) with various branching parameters (σ). (**c**) Distribution of burst prediction scores obtained from pooling 100 branching models (σ = 1). (**d**) Scatter plot of firing rates versus burst prediction scores (DIV 17).

**Figure 5:**
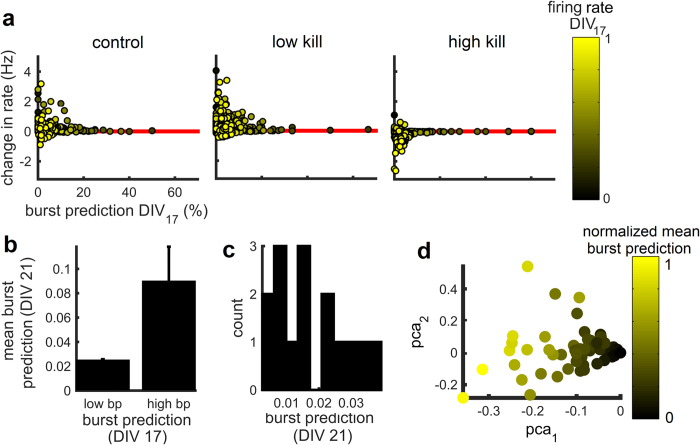
Strong burst predictors remain stable after insult. (**a**) Burst prediction scores versus change in firing rate (DIV 17 vs. 21) for one representative culture of control, low kill, and high kill conditions. Firing rates were normalized between [0,1]. Each dot corresponds to a single channel. (**b**) Burst prediction scores of low kill cultures pre- (DIV 17) and post- (DIV 21) treatment. High BP, electrodes with the top five scores. Low BP, all other electrodes. Vertical bars, SEM. (**c**) Burst prediction scores obtained at DIV 21 for electrodes whose scores at DIV 17 was zero. (**d**) Principal components analysis of burst prediction scores across 5 days *in vitro* (DIV 15, 16, 17, 21, and 22) for a representative culture of the low kill condition.

Neuronal injury was assessed in two stages. First, at DIV 18 prior to insult application, phase contrast images that illuminated healthy cell bodies were acquired and neurons visible within the electrode field were manually counted, in order to provide a live neuron count (Fig. 2a, top row). Second, at 19 DIV, 24 h following neurotoxin exposure, cultures were exposed to the cell death marker propidium iodide (PI; 4.5 μM) that is membrane impermeable, and markedly increases its fluorescence by binding to the DNA of neurons whose plasma membrane has fragmented (Fig. 2a, bottom row)^13^. PI-positive fluorescent cells were manually counted using images obtained on a fluorescence microscope (Zeiss Axiovert 200 with Lambda DG-4). Images were processed using the ImageJ environment (National Institute of Health). The percentage of dead neurons was determined as the ratio of PI-positive neurons measured at 19 DIV (24 h post-insult) to the number of phase-bright neurons measured at 18 DIV (immediately prior to insult), and is presented as the mean ± standard error for each experimental condition (control [*N* = 9], low kill [*N* = 14], high kill [*N* = 7]). Statistical comparisons were made by ANOVA, with statistical significance inferred at *p* < 0.05.

## Additional Information

**How to cite this article**: Kuebler, E. S. *et al.* Burst predicting neurons survive an *in vitro* glutamate injury model of cerebral ischemia. *Sci. Rep.* **5**, 17718; doi: 10.1038/srep17718 (2015).

## Supplementary Material

Supplementary Information

## References

[b1] Wagenaar D. A., Pine J. & Potter S. M. An extremely rich repertoire of bursting patterns during the development of cortical cultures. BMC Neurosci 7, 11 (2006). 16464257 10.1186/1471-2202-7-11 PMC1420316

[b2] Kepecs A. & Lisman J. Information encoding and computation with spikes and bursts. Network 14, 103–118 (2003). 12613553

[b3] Kepecs A., Wang X. J. & Lisman J. Bursting neurons signal input slope. J Neurosci 22, 9053–9062 (2002). 12388612 10.1523/JNEUROSCI.22-20-09053.2002 PMC6757694

[b4] Butera R. J. Jr. Rinzel J. & Smith J. C. Models of respiratory rhythm generation in the pre-Botzinger complex. I. Bursting pacemaker neurons. J Neurophysiol 82, 382–397 (1999). 10400966 10.1152/jn.1999.82.1.382

[b5] Beggs J. M. The criticality hypothesis: how local cortical networks might optimize information processing. Phil Trans A, 366, 329–343 (2008). 10.1098/rsta.2007.2092 17673410

[b6] Touboul J. & Destexhe A. Can power-law scaling and neuronal avalanches arise from stochastic dynamics? PLoS One 5, e8982 (2010). 20161798 10.1371/journal.pone.0008982 PMC2820096

[b7] Eytan D. & Marom S. Dynamics and effective topology underlying synchronization in networks of cortical neurons. J Neurosci 26, 8465–8476 (2006). 16914671 10.1523/JNEUROSCI.1627-06.2006 PMC6674346

[b8] Eckmann J.-P., Jacobi S., Marom S., Moses E. & Zbinden C. Leader neurons in population bursts of 2D living activity. New J Phys 10, 19 (2008).

[b9] Zbinden C. *Leader neurons in living neural networks and in leaky integrate and fire neuron models*, PhD thesis, University of Geneve (2010).

[b10] Zbinden C. Leader neurons in leaky integrate and fire neural network simulations. J Comput Neurosci 31, 285–304 (2011). 21234795 10.1007/s10827-010-0308-6

[b11] Bonifazi P. *et al.* GABAergic hub neurons orchestrate synchrony in developing hippocampal networks. Science 326, 1419–1424 (2009). 19965761 10.1126/science.1175509

[b12] Schwartz-Bloom R. D. & Sah R. gamma-Aminobutyric acid(A) neurotransmission and cerebral ischemia. J Neurochem 77, 353–371 (2001). 11299298 10.1046/j.1471-4159.2001.00274.x

[b13] Tauskela J. S. *et al.* Elevated synaptic activity preconditions neurons against an *in vitro* model of ischemia. J Biol Chem 283, 34667–34676 (2008). 18845540 10.1074/jbc.M805624200 PMC3259903

[b14] Meldrum B. S. Glutamate as a neurotransmitter in the brain: review of physiology and pathology. J Nutrit 130, 1007S–1015S (2000). 10736372 10.1093/jn/130.4.1007S

[b15] Vincent K., Tauskela J. S. & Thivierge J. P. Extracting functionally feedforward networks from a population of spiking neurons. Front Comput Neurosci 6, 86 (2012). 23091458 10.3389/fncom.2012.00086 PMC3476068

[b16] Vincent K., Tauskela J. S., Mealing G. A. & Thivierge J. P. Altered network communication following a neuroprotective drug treatment. PLoS One 8, e54478 (2013). 23349901 10.1371/journal.pone.0054478 PMC3551770

[b17] Thivierge J. P. Scale-free and economical features of functional connectivity in neuronal networks. Phys Rev E Stat Nonlin Soft Matter Phys 90, 022721 (2014). 25215772 10.1103/PhysRevE.90.022721

[b18] Langlois D., Cousineau D. & Thivierge J. P. Maximum likelihood estimators for truncated and censored power-law distributions show how neuronal avalanches may be misevaluated. Phys Rev E Stat Nonlin Soft Matter Phys 89, 012709 (2014). 24580259 10.1103/PhysRevE.89.012709

[b19] Beggs J. M. & Plenz D. Neuronal avalanches in neocortical circuits. J Neurosci 23, 11167–11177 (2003). 14657176 10.1523/JNEUROSCI.23-35-11167.2003 PMC6741045

[b20] Bauke H. Parameter estimation for power-law distributions by maximum likelihood methods. Eur Phys J B 58, 167–173 (2007).

[b21] Velandia-Romero M. L., Castellanos J. E. & Martinez-Gutierrez M. *In vivo* differential susceptibility of sensory neurons to rabies virus infection. J Neurovirol 19, 367–375 (2013). 10.1007/s13365-013-0179-5 23959650

[b22] Cho H. *et al.* Differential innate immune response programs in neuronal subtypes determine susceptibility to infection in the brain by positive-stranded RNA viruses. Nat Med 19, 458–464 (2013). 23455712 10.1038/nm.3108 PMC3618596

[b23] Srinivas K. V., Jain R., Saurav S. & Sikdar S. K. Small-world network topology of hippocampal neuronal network is lost, in an *in vitro* glutamate injury model of epilepsy. Eur J Neurosci 25, 3276–3286 (2007). 17552996 10.1111/j.1460-9568.2007.05559.x

[b24] Blau A., Neumann T., Ziegler C. & Benfenati F. Replica-moulded polydimethylsiloxane culture vessel lids attenuate osmotic drift in long-term cell cultures. J Biosci 34, 59–69 (2009). 19430119 10.1007/s12038-009-0009-3

[b25] Quian Quiroga R. & Panzeri S. Extracting information from neuronal populations: information theory and decoding approaches. Nat Rev Neurosci 10, 173–185 (2009). 19229240 10.1038/nrn2578

[b26] Quiroga R. Q., Nadasdy Z. & Ben-Shaul Y. Unsupervised spike detection and sorting with wavelets and superparamagnetic clustering. Neural Comput 16, 1661–1687 (2004). 15228749 10.1162/089976604774201631

[b27] Gao J. *et al.* Isoform-specific stimulation of cardiac Na/K pumps by nanomolar concentrations of glycosides. J Gen Physiol 119, 297–312 (2002). 11929882 10.1085/jgp.20028501 PMC2238186

[b28] Bonde C. *et al.* Neurotoxic and neuroprotective effects of the glutamate transporter inhibitor DL-threo-beta-benzyloxyaspartate (DL-TBOA) during physiological and ischemia-like conditions. Neurochem Internat 43, 371–380 (2003). 10.1016/s0197-0186(03)00024-x 12742081

[b29] Tauskela J. S., Aylsworth A., Hewitt M., Brunette E. & Mealing G. A. Preconditioning induces tolerance by suppressing glutamate release in neuron culture ischemia models. J Neurochem 122, 470–481 (2012). 22607164 10.1111/j.1471-4159.2012.07791.x

