# Supplementary material for: Burst predicting neurons survive an in vitro glutamate injury model of cerebral ischemia
Source: Sci Rep. 2015 Dec 9;5:17718. doi: 10.1038/srep17718 (PMC4673430; doi:10.1038/srep17718)
Supplement: Supplementary Information [file srep17718-s1.doc]

**Supplementary Material**

**Burst predicting neurons survive an *in vitro* glutamate injury model of cerebral ischemia**

**Eric S. Kuebler^1^, Joseph S. Tauskela^2^**, **Amy Aylsworth^2^, Xigeng Zhao^2^, &** **Jean-Philippe Thivierge^1†^**

^1^Center for Neural Dynamics and School of Psychology, University of Ottawa, Ottawa, Ontario, Canada.

^2^Department of Translational Bioscience, Human Health Therapeutics, National Research Council of Canada, Ottawa, Ontario, Canada.

*Timing of Burst Predictor Activity*

Experimental work shows that the interval between cells firing prior to the onset of a burst is shorter in mature compared to immature cultures [^21^](#_ENREF_21). Because our recordings took place over several days *in vitro*, this effect may influence our ability to detect burst predictors. To examine this possibility, we measured the interval between each of the top five burst predicting channels (DIV 17 and 21 of controls) and the onset of bursts (Fig. S1a). Cultures at DIV 21 had shorter intervals than DIV 17, with a distribution of intervals that was positively skewed (Fig. S1b). While these distributions were not significantly different (Wilcoxon rank test, *n* = 347, *p* > .423), it is worth considering how changes in the timing of burst predictors would affect our results. These changes would be particularly concerning if, instead of intervals becoming shorter, these intervals became longer over the course of maturation. In this case, strong burst predictors identified at DIV 17 might fall outside of our pre-burst time window (50 ms) at DIV 21. However, if intervals become shorter (as shown here), strong predictors identified at DIV 17 would still fall within the same 50 ms time window at DIV 21 (albeit with an activation that was closer to burst onset). Burst predictors that might emerge at DIV 21 due to shorter time intervals than DIV 17 are not considered in the above analyses, where all burst predictor scores are computed at DIV 17.

*Spatial Distribution of Burst Predictors*

In a final series of analyses, we examined whether strong burst predictors were spatially clustered on the recording array. First, we identified the channels with the top five BP scores (Fig. S2a). Then, we measured the Euclidean distance between each pair of these channels (inter-electrode distance, 200 *µ*m). The distribution of distances taken over all cultures at baseline (DIV 17) followed a broad distribution with a mean of 738.36 *µ*m (s.d., 177.6 *µ*m) (Fig. S2b). To determine if strong burst predictors were clustered on the array, we computed a Pearson correlation between mean BP score and physical distance between pairs of channels. The lack of a strong relationship (*r*^2^(35) = .01, *p* > .958) shows that strong predictors were not systematically clustered in space, but instead were broadly distributed across the array.

**
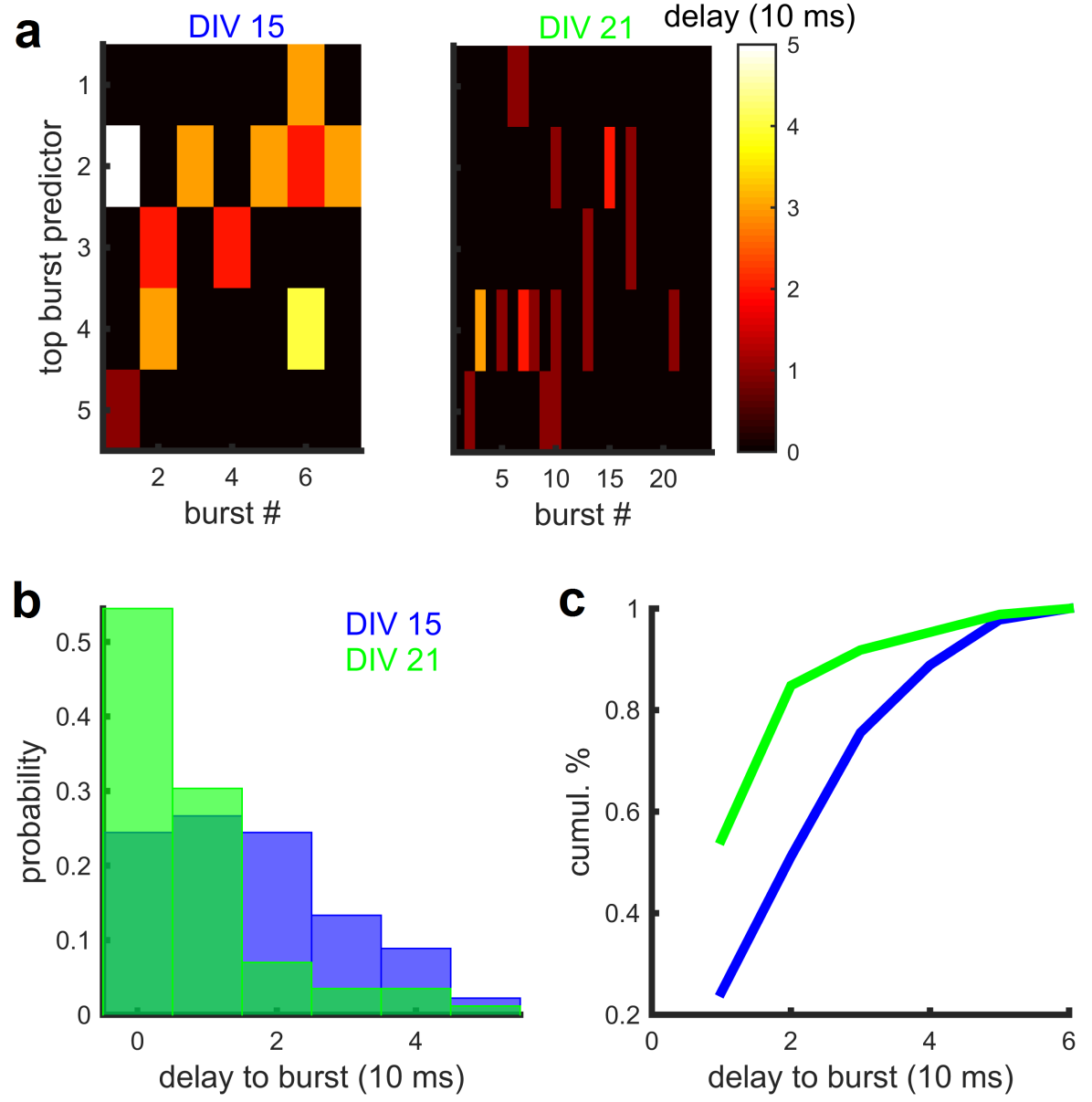

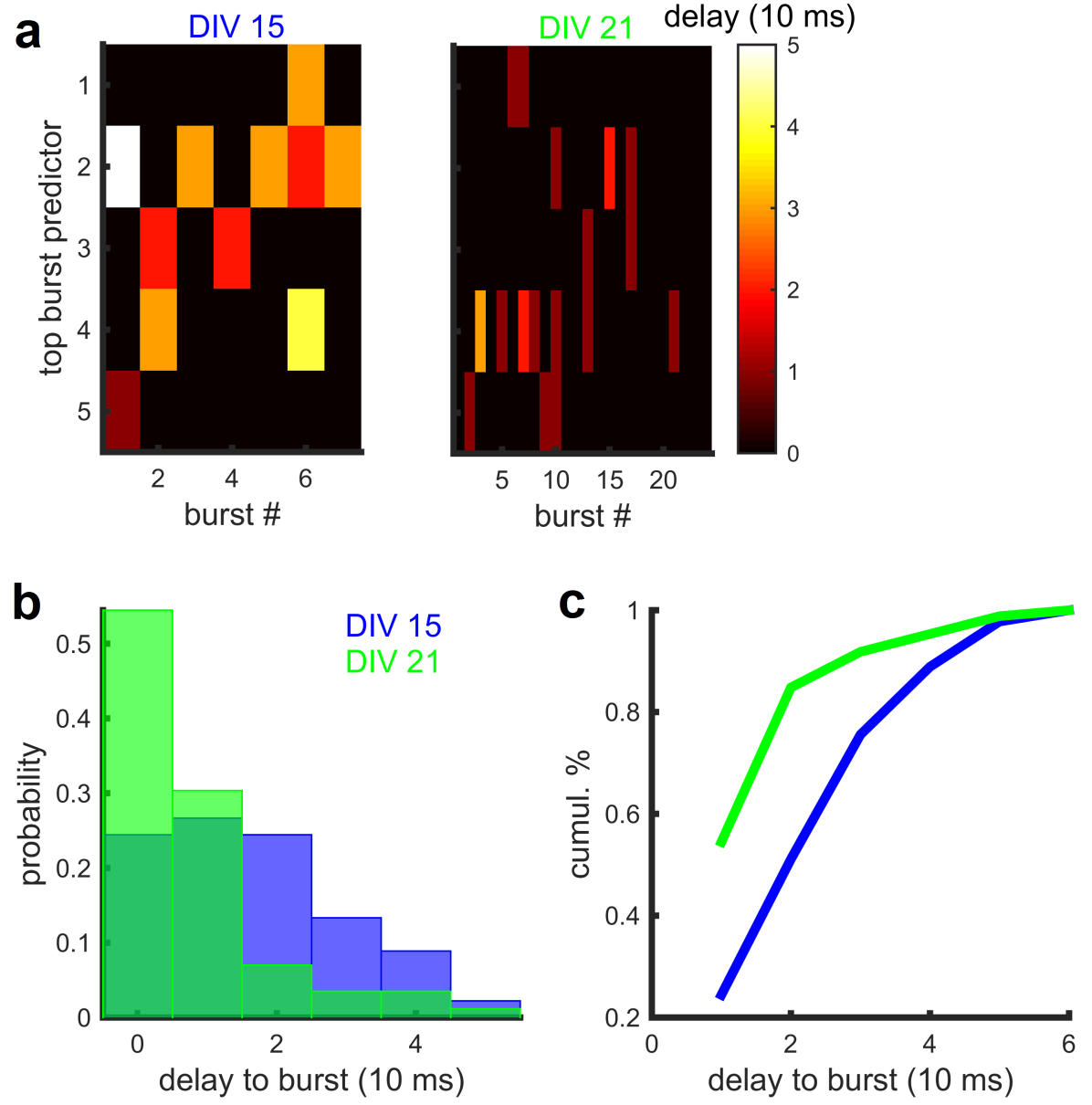
**

**Figure S1. Time interval between the activity of burst predictors and burst onsets**. **a**. Heat map of the time interval (in ms) between the five strongest burst predictors and network burst onset in one representative control culture (DIV 15 and 21). **b**. Distributions of time intervals for control cultures (DIV 15 and 21).

**
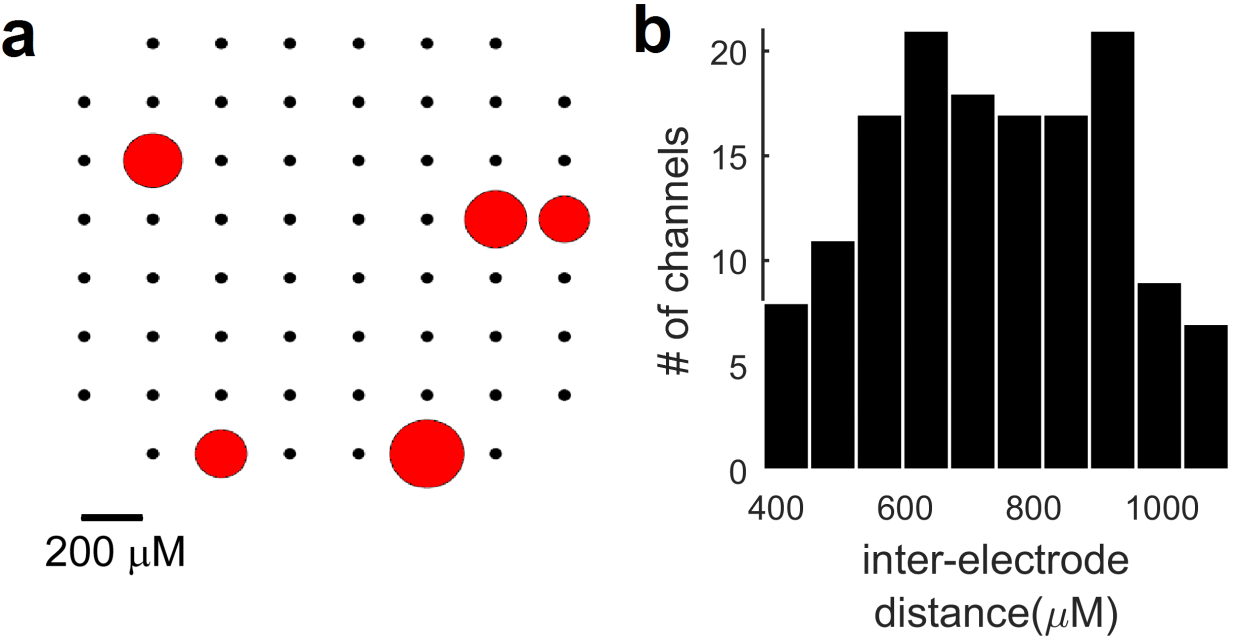
**

**Figure S2. Spatial distribution of the five strongest burst predictors. a.** Example MEA with five strongest burst predictors (diameter of circles corresponding to value of BP score) and their spatial location on the MEA (black circles). **b**. Histogram of physical distances (measured in *µ*m) between all pairs of the five strongest burst predictors across all MEA of the control condition (DIV 17).
